# Comparison of the tuberculin skin test and the QuantiFERON-TB Gold test in detecting latent tuberculosis in health care workers in Iran

**DOI:** 10.4178/epih.e2016032

**Published:** 2016-07-24

**Authors:** Ehsan Mostafavi, Mahshid Nasehi, Abdolrazagh Hashemi Shahraki, Saber Esmaeili, Ebrahim Ghaderi, Saeed Sharafi, Amin Doosti-Irani

**Affiliations:** 1 Department of Epidemiology, Pasteur Institute of Iran, Tehran, Iran; 2 Research Centre for Emerging and Reemerging Infectious Diseases, Pasteur Institute of Iran, Akanlu, Kabudar-Ahang, Hamadan, Iran; 3 Center for Diseases Control and Prevention, Ministry of Health and Medical Education, Tehran, Iran; 4 Department of Epidemiology and Biostatistics, School of Public Health, Iran University of Medical Sciences, Tehran, Iran; 5 Department of Bacteriology, Faculty of Medical Sciences, Tarbiat Modares University, Tehran, Iran; 6 Social Determinants of Health Research Center, Kurdistan University of Medical Sciences, Sanandaj, Iran; 7 Department of Epidemiology and Biostatistics, School of Medicine, Kurdistan University of Medical Sciences, Sanandaj, Iran; 8 Department of Epidemiology and Biostatistics, School of Public Health, Tehran University of Medical Sciences, Tehran, Iran

**Keywords:** Latent tuberculosis, Tuberculin skin test, QuantiFERON-TB Gold, Health care workers

## Abstract

**OBJECTIVES:**

The tuberculin skin test (TST) and the QuantiFERON-TB Gold test (QFT) are used to identify latent tuberculosis infections (LTBIs). The aim of this study was to determine the agreement between these two tests among health care workers in Iran.

**METHODS:**

This cross-sectional study included 177 tuberculosis (TB) laboratory staff and 67 non-TB staff. TST indurations of 10 mm or more were considered positive. The Student’s t-test and the chi-square test were used to compare the mean score and proportion of variables between the TB laboratory staff and the non-TB laboratory staff. Kappa statistics were used to evaluate the agreement between these tests, and logistic regression was used to assess the risk factors associated with positive results for each test.

**RESULTS:**

The prevalence of LTBIs according to both the QFT and the TST was 17% (95% confidence interval [CI], 12% to 21%) and 16% (95% CI, 11% to 21%), respectively. The agreement between the QFT and the TST was 77.46%, with a kappa of 0.19 (95% CI, 0.04 to 0.34).

**CONCLUSIONS:**

Although the prevalence of LTBI based on the QFT and the TST was not significantly different, the kappa statistic was low between these two tests for the detection of LTBIs.

## INTRODUCTION

It is estimated that one-third of the world’s population currently has a latent tuberculosis infection (LTBI) as a result of infection with *Mycobacterium tuberculosis* [1]. It is estimated that approximately 10% of individuals with an LTBI may develop active tuberculosis (TB) during their lifetime [2]. The timely detection of LTBIs is important to prevent the development of active TB [3].

The tuberculin skin test (TST) is the most commonly used test to identify LTBIs. Although the TST is inexpensive and simple, facilitating its use, especially in developing countries, the validity and reliability of the TST is affected by many factors, including Bacillus Calmette-Guérin (BCG) vaccination, infection with non-TB *Mycobacterium* (NTM) species, the method of TST administration, the interpretation of the reaction, insufficient dosage, and cutaneous anergy [4-7].

In recent years, the QuantiFERON-TB Gold test (QFT), which is one of the newly developed interferon-gamma release assays (IGRAs) used to diagnose TB infections, has been used extensively for the detection of latent TB [8]. The test identifies the level of interferon (IFN)-gamma produced in reaction to * M. tuberculosis*-specific antigens [9]. IGRAs have several important advantages over the TST. The most important advantage of IGRAs is that, unlike TST, they are not influenced by factors such as BCG vaccination, most NTM species, or antigen dose [8,10]. Nevertheless, IGRAs have some important disadvantages, such as involving additional material costs and requiring a well-equipped laboratory and blood sampling with subsequent special handling to preserve the viability of lymphocytes [8]. However, IGRAs have a high specificity and a similar sensitivity to the TST for the detection of LTBIs [10,11].

Studies have shown poor agreement between IGRAs and the TST [12-14]. However, in countries with a high incidence of TB, an acceptable level of agreement has been reported between the TST and IGRA. In contrast, studies conducted in countries with an annual incidence of TB of ≤20 per 100,000 have found poor agreement between the QFT and the TST [15]. More evidence is therefore needed to evaluate discrepancies in the results between IGRAs and the TST [10].

Given the lack of evidence regarding instances of disagreement between the TST and IGRAs in the detection of LTBIs among health care workers (HCWs) in Iran, the aim of this study was to determine the agreement between these two tests among Iranian HCWs.

## MATERIALS AND METHODS

A proposal for this study was approved by the scientific committee of the Pasteur Institute of Iran. This cross-sectional study was performed between November 2013 and January 2014 in eight universities of the medical sciences, including Shiraz, Golestan, Shahid Beheshti, Iran, Tehran, Tabriz, Kermanshah, and Isfahan. All of these universities contain regional reference TB laboratories performing microscopic examinations, cultures, and drug sensitivity testing on * M. tuberculosis* isolates from throughout Iran.

In this study, all TB laboratory staff (177 participants) and a random sample of non-TB staff (67 participants), including administrative, finance, and service staff were included. Pregnant women were excluded from this study. A questionnaire was used to gather information regarding demographic variables, place and history of work, history of contact with TB patients, the presence of a BCG vaccination scar, and history of purified protein derivative skin tests.

Trained staff injected 0.1 mL (5 tuberculin units) of human tuberculin (Razi Vaccine and Serum Research Institute, Karaj, Iran) intradermally into the dorsal or volar surface of the forearm. The TST response after 48 to 72 hours was read by a trained technician. TST reactions of ≥10 mm were considered positive, while reactions of <10 mm were interpreted as negative [6].

The QFT was performed using a blood sample. Blood samples were obtained before the TST. At least 3 mL of blood was obtained from each participant, and then a 1-mL blood sample was transferred into each of three QuantiFERON-TB Gold tubes (Cellestis Ltd., Victoria, Australia). Blood samples with test antigens were incubated for 16 to 24 hours based on the kit instructions. The plasma samples were harvested into new labeled tubes and were delivered to the Pasteur Institute of Iran through a 4°C cold chain. The concentration of IFN-gamma (IU/mL) was measured using an automated enzyme-linked immunosorbent assay. The results of the test were interpreted using software supplied by the manufacturer (Cellestis Ltd.) including a cut-off point for the detection of IFN-gamma.

The individuals who had positive TST or QFT results were visited by an expert specialist physician. Active TB was diagnosed based on clinical findings, such as general symptoms (including fatigue, malaise, fever, weight loss, and anorexia) and chronic, productive cough with purulent sputum, in combination with a chest X-ray with radiological features consistent with TB disease.

The prevalence of LTBIs was estimated with 95% confidence intervals (CIs) using the QFT and TST. The concordance between these two tests was evaluated using proportion agreement and the kappa (κ) statistic. The κ statistic was interpreted as follows: κ>0.75 was considered to indicate excellent agreement, κ<0.40 to indicate poor agreement, and κ between 0.40 and 0.75 to indicate fair to good agreement [16].

Both the Student’s t-test and the chi-square test were used to compare means and proportions of variables between the TB laboratory staff and the non-TB laboratory staff. Logistic regression was used to identify risk factors associated with positive results for each test. Unadjusted and adjusted odds ratios (ORs) were reported to assess the effects of covariates on LTBI incidence. All statistical analyses were conducted using Stata version 11.0 (Stata Corp., College Station, TX, USA) and the results were reported with 95% CIs.

## RESULTS

In this study, 244 participants, including 177 TB laboratory staff and 67 non-TB staff, were assessed for LTBIs using both the TST and QFT. The mean age of the TB lab staff and the non-TB staff was 36.75 years (standard error [SE], 0.54) and 39.40 years (SE, 1.15), respectively. Males comprised 60.25% (147 of 244) of the participants (Table 1).

The total estimated prevalence of LTBIs based on the TST was 16% (95% CI, 11% to 21%). The prevalence of LTBIs based on the TST among males was significantly higher than among females (p<0.05). Additionally, the prevalence of LTBIs based on this test increased with age, as subjects ≥50 years old had a higher prevalence of LTBIs (p<0.05) (Table 2).

Logistic regression analysis showed the adjusted OR for TB lab staff in comparison to non-TB staff to be 0.31 (95% CI, 0.11 to 0.93). The adjusted OR for participants who had a history of contact with TB patients was 1.70 (95% CI, 0.71 to 4.05), and positive TST results among participants with a history of BCG vaccination were more common than among those with no such history (OR, 2.23; 95% CI, 0.67 to 7.43) (Table 3).

The difference in the prevalence of LTBIs measured using the QFT was not statistically significant across any subgroups. The total estimated prevalence of LTBIs based on the QFT was 17% (95% CI, 12% to 21%). The prevalence of LTBIs among TB lab staff and non-TB staff was 19% (95% CI, 12% to 24%) and 13% (95% CI, 5% to 23%), respectively (Table 2).

According to the adjusted logistic regression, subjects with a history of work of 20 years or more were more likely to have positive QFT results than subjects with a history of work of zero to four years (OR, 2.64; 95% CI, 0.53 to 13.09). Participants with a history of BCG vaccination were less likely to be QFT-positive than others (OR, 0.50; 95% CI, 0.21 to 1.23) (Table 3).

The overall agreement and κ statistic were 77.46% and 0.19 (95% CI, 0.04 to 0.34), respectively. Both the TST and QFT were positive in 13 and negative in 176 subjects (Table 4).

## DISCUSSION

The results of this study showed that the estimated prevalence of LTBIs according to the QFT and the TST was 17% and 16%, respectively. The estimated value of agreement between the QFT and the TST was 77.46%, and the κ statistic was 0.19 (95% CI, 0.04 to 0.34).

The logistic regression analysis indicated different adjusted ORs associated with the QFT and the TST. The adjusted OR for people with ≥20 years of work history was 2.64 times the baseline for the QFT and 0.54 times the baseline for the TST. These results were, however, not statistically significant due to the low sample size of this subgroup.

Another study among Iranian HCWs reported agreement and a κ statistic of 73.8% and 0.39 between the tests, respectively [17]. The agreement between the two tests (TST and QFT) was very similar in that study and our study, but the κ statistic was higher in their study. This discrepancy may reflect differences in the study population. The prevalence of TB in different regions of Iran is not the same [18-21], and the κ statistic is influenced by the prevalence of a disease [22]. In the previous study [17], all subjects were HCWs in a hyper-endemic region. Although the overall agreement between the two tests was good, it is important to note that the measures of agreement have an important limitation; namely, these measures do not take into account the possibility that agreement may occur by chance alone [16].

In another study among HCWs in France, the agreement between the QFT and the TST was weak, and the κ value was 0.11 [23]. In a study of HCWs in Turkey, the overall agreement and κ statistic were 63.1% and 0.22, respectively [24]. The κ statistic between the QFT and the TST was found to be 0.22 in South Korean HCWs [25]. Among HCWs in the US, the agreement and κ value between the QFT and TST were 63.2% and 0.31 (95% CI, 0.27 to 0.35), respectively [26]. The κ value between the QFT and TST in these studies [17,23-26] is almost the same as was found in our study and indicates poor agreement between the QFT and the TST.

According to the results of a meta-analysis, the κ statistic between the QFT and TST among HCWs in countries with a high burden of TB (0.38) was significantly more than has been observed in low-burden countries (0.21) [27]. Therefore, it might be concluded that the agreement of the QFT and TST in populations with a high risk of TB infection seems to be better than in low-risk populations. However, in the high-risk population investigated in the current study, the agreement was also found to be poor. The differences in the κ statistic among various studies may be due to differences in the prevalence of LTBIs in various study populations. Moreover, one of the limitations of the κ statistic is its dependence on prevalence; namely, the κ statistic is higher in populations in which the prevalence is high than in populations with a lower prevalence [16].

One study [17] showed that a family history of TB was a potential risk factor for LTBI based on the QFT (OR,7.96) and TST (OR, 4.91). We did not observe any analogous findings. In our study, the adjusted OR for contact with TB patients was 0.73 (95% CI, 0.34 to 1.56) for the QFT and 1.70 (95% CI, 0.71 to 4.05) for the TST. One reason for this may be the availability and use of suitable protection while working with patients and/or their sputum samples. In the patients’ family members, contact was not found to be protective.

BCG vaccination had a non-significant protective association with LTBIs based on the QFT (OR, 0.50; 95% CI, 0.21 to 1.23), while the association of BCG vaccination with LTBI based on the TST was not significant (OR, 2.23; 95% CI, 0.67 to 7.43). This inconsistency may be due to the influence of BCG vaccination on the results of the TST, since other studies have shown that the results of the TST were influenced by BCG vaccination [8,10]. In a study among HCWs in the US, a positive TST with a negative QFT was associated with BCG vaccination (OR, 25.1; 95% CI, 15.5 to 40.5) [26].

This study was conducted in eight universities of medical science in Iran. All QFT samples were sent to the Department of Epidemiology of the Pasteur Institute of Iran, and the tests were performed by a trained lab expert. The TST tests were conducted by different lab experts and technicians in the eight universities. Although we trained all lab experts and technicians in the method of TST testing, heterogeneity may exist in TST testing by multiple lab experts and technicians, so this factor may have affected the results of the TST test.

The prevalence of LTBIs according to both the QFT and TST was considerable. Although the prevalence of LTBIs based on the QFT and TST was not significantly different, and the overall agreement between both tests was good, the κ statistic was low between these two tests. The κ value among the non-BCG vaccinated group was higher than among the vaccinated group. It seems the use of the QFT test, due to its high cost, is only appropriate in cases where the results of a TST could be affected by BCG vaccination or NTM species.

## Figures and Tables

**Table 1.** Demographic characteristics of the participants

	TB lab staff		Non-TB staff		p-value	Total
	n	%	n	%		
Sex						
Male	100	56.50	47	70.15	0.05	147
Female	77	43.50	20	29.85		97
Age (yr)						
20-29	29	15.85	10	14.93	0.31	39
30-39	86	46.99	26	38.81		112
40-49	56	30.60	22	32.84		78
≥50	12	6.56	9	123.43		21
Education level						
No high school diploma	17	9.60	12	17.91	0.001	29
High school diploma	13	7.34	30	44.78		43
Associate degree	59	33.33	8	11.94		67
BS	74	41.81	16	23.88		90
Master of science and above	14	7.91	1	1.49		15
Job						
Lab position requiring a BS	85	48.02	-	-	0.001	85
Technician	60	33.90	-	-		60
Service personnel in lab	32	18.08	-	-		32
Administrative staff	-	-	42	62.69		42
Finance staff	-	-	10	14.93		10
Service personnel	-	-	15	22.39		15
History of work (yr)						
0-4	62	35.03	14	20.90	0.001	76
5-9	42	23.73	10	14.93		52
10-14	36	20.34	12	17.91		48
15-19	25	14.12	13	19.40		38
≥20	12	6.78	18	26.87		30
History of BCG vaccination	159	89.83	50	74.63		209

TB, tuberculosis; BS, bachelor of science; BCG, Bacillus Calmette-Guérin.

**Table 2.** Comparison of the prevalence of LTBIs according to the QFT and TST

Variable	QFT		p-value	TST		p-value	p-value^1^
	Prevalence	95% CI		Prevalence	95% CI		
Sex							
Male	0.17	0.11, 0.23	0.92	0.20	0.13, 0.26	<0.05	0.46
Female	0.18	0.10, 0.25		0.10	0.04, 0.16		0.22
Age (yr)							
20-29	0.10	0.03, 0.24	0.13	0.03	0.00, 0.13	<0.05	0.16
30-39	0.17	0.10, 0.25		0.16	0.10, 0.25		0.86
40-49	0.23	0.14, 0.34		0.22	0.13, 0.33		0.85
≥50	0.05	0.00, 0.24		0.23	0.08, 0.47		0.07
Education level							
No high school diploma	0.21	0.06, 0.36	0.68	0.28	0.11, 0.44	0.49	0.54
High school diploma	0.09	0.00, 0.18		0.12	0.02, 0.21		0.50
Associate degree	0.18	0.09, 0.27		0.16	0.07, 0.25		0.82
BS	0.18	0.10, 0.26		0.13	0.06, 0.20		0.54
Master of science and above	0.27	0.03, 0.50		0.20	0.01, 0.41		0.67
Job group							
TB lab staff	0.19	0.12, 0.24	0.34	0.14	0.09, 0.19	0.20	0.25
Non-TB staff	0.12	0.05, 0.22		0.21	0.11, 0.31		0.39
Job							
Lab position requiring a BS	0.19	0.10, 0.27	0.78	0.14	0.07, 0.22	0.58	0.54
Technician	0.17	0.07, 0.26		0.17	0.07, 0.26		0.81
Service personnel in lab	0.22	0.07, 0.37		0.09	0.01, 0.20		0.17
Administrative staff	0.14	0.04, 0.25		0.19	0.07, 0.31		0.56
Finance staff	-	-		0.10	0.09, 0.30		0.31
Service personnel	0.20	0.01, 0.41		0.33	0.09, 0.58		0.41
History of work (yr)							
0-4	0.16	0.07, 0.24	0.42	0.11	0.03, 0.18	0.46	0.34
5-9	0.15	0.05, 0.24		0.13	0.04, 0.22		0.78
10-14	0.19	0.07, 0.30		0.21	0.09, 0.33		0.62
15-19	0.16	0.04, 0.28		0.18	0.06, 0.31		0.56
≥20	0.23	0.08, 0.38		0.22	0.08, 0.39		1.00
BCG vaccination							
No	0.26	0.11, 0.40	0.15	0.11	0.01, 0.22	0.42	0.12
Yes	0.16	0.11, 0.21		0.17	0.12, 0.22		0.79
Total	0.17	0.12, 0.21		0.16	0.11, 0.21		0.90

LBTI, latent tuberculosis infection; TST, tuberculin skin test, QFT, QuantiFERON-TB Gold test; CI, confidence interval; TB, tuberculosis; BS, bachelor of science; BCG, Bacillus Calmette-Guérin.

1 For the comparison of the TST and QFT in each subgroup.

**Table 3.** Results of the TST and QFT according to probable risk factors

Variable	QF				TST			
	Unadjusted	p-value	Adjusted	p-value	Unadjusted	p-value	Adjusted	p-value
Sex								
Female	1.00		1.00		1.00		1.00	
Male	0.96 (0.49, 1.90)	0.89	0.98 (0.46, 2.07)	0.94	2.14 (0.99, 4.61)	0.05	1.75 (0.75, 4.08)	0.20
Age	1.01 (0.97, 1.05)	0.69	0.99 (0.92, 1.06)	0.77	1.07 (1.02, 1.11)	0.003	1.07 (1.00, 1.15)	0.05
Education Level								
No high school diploma	1.00		1.00		1.00		1.00	
High school diploma	0.39 (0.10, 1.54)	0.15	0.43 (0.10, 1.87)	0.26	0.35 (0.10, 1.19)	0.09	0.40 (0.10, 1.58)	0.19
Associate degree	0.84 (0.28, 2.49)	0.70	0.81 (0.22, 2.95)	0.75	0.52 (0.18, 1.46)	0.21	1.27 (0.35, 4.55)	0.72
Bachelor of science	0.83 (0.29, 2.36)	0.69	0.79 (0.22, 2.80)	0.71	0.40 (0.15, 1.12)	0.8	1.02 (0.28, 3.70)	0.98
Master of science and above	1.40 (0.33, 5.97)	0.66	1.19 (0.23, 6.10)	0.84	0.66 (0.15, 2.95)	0.58	1.75 (0.30, 10.16)	0.53
Job group								
Non-lab staff	1.00		1.00		1.00		1.00	
TB lab staff	1.48 (0.67, 3.28)	0.34	1.88 (0.67, 5.29)	0.23	0.62 (0.30, 1.26)	0.20	2.23 (0.11, 0.93)	0.04
History of work (yr)								
0-4	1.00		1.00		1.00		1.00	
5-9	0.97 (0.37, 2.57)	0.95	1.00 (0.36, 2.74)	0.99	1.32 (0.45, 3.90)	0.61	1.07 (0.35, 3.30)	0.9
10-14	1.23 (0.47, 3.18)	0.67	1.42 (0.49, 4.13)	0.52	2.24 (0.81, 6.14)	0.12	1.24 (0.41, 3.75)	0.71
15-19	1.00 (0.34, 2.90)	1.00	1.30 (0.36, 4.61)	0.69	1.92 (0.64, 5.76)	0.25	0.77 (0.21, 2.85)	0.69
≥20	1.62 (0.57, 4.62)	0.36	2.64 (0.53, 13.09)	0.24	2.58 (0.84, 7.92)	0.09	0.54 (0.11, 2.68)	0.45
Contact with TB patients								
No	1.00		1.00		1.00		1.00	
Yes	0.95 (0.49, 1.85)	0.87	0.73 (0.34, 1.56)	0.42	1.43 (0.72, 2.84)	0.31	1.70 (0.71, 4.05)	0.23
BCG vaccination								
No	1.00		1.00		1.00		1.00	
Yes	0.54 (0.23, 1.26)	0.15	0.50 (0.21, 1.23)	0.13	1.56 (0.52, 4.70)	0.43	2.23 (0.67, 7.43)	0.19

Values are presented as odds ratio (95% confidence interval). TST, tuberculin skin test, QFT, QuantiFERON-TB Gold test; TB, tuberculosis; BCG, Bacillus Calmette-Guérin.

**Table 4.** Overall agreement and kappa values between the QFT and TST results

Group	TST results (cut-off point of ≥ 10 mm)	QFT results		Total	Agreement (%)	Kappa (95%CI)	p-value
		Negative	Positive				
TB staff	Negative	128	24	152	77.40	0.18 (0.00, 0.35)	0.008
	Positive	16	9	25			
	Total	144	33	177			
Non-TB staff	Negative	48	5	53	77.61	0.22 (-0.06, 0.50)	0.03
	Positive	10	4	14			
	Total	58	9	67			
BCG vaccinated	Negative	150	22	172	76.92	0.16 (-0.00, 0.32)	0.01
	Positive	26	10	36			
	Total	176	32	208			
Non-BCG vaccinated	Negative	25	7	32	75.00	0.18 (-0.16, 0.53)	0.11
	Positive	2	2	4			
	Total	27	9	36			
All participants	Negative	176	29	205	77.46	0.19 (0.04, 0.34)	0.001
	Positive	26	13	39			
	Total	202	33	244			

QFT, QuantiFERON-TB Gold test; TST, tuberculin skin test; CI, confidence interval; TB, tuberculosis; BCG, Bacillus Calmette-Guerin.

## References

[1] World Health Organization (2012). Tuberculosis: fact sheet no. 104. http://www.who.int/mediacentre/factsheets/who104/en/print.html.

[2] Comstock GW, Livesay VT, Woolpert SF (1974). The prognosis of a positive tuberculin reaction in childhood and adolescence. Am J Epidemiol.

[3] Fitzgerald DW, Sterling TR, Has DW, Mandell GL, Douglas RG, Bennett JE, Dolin R, Blaser MJ (2010). Mycobacterium tuberculosis. Mandell, Douglas, and Bennett’s principles and practice of infectious diseases.

[4] Farhat M, Greenaway C, Pai M, Menzies D (2006). False-positive tuberculin skin tests: what is the absolute effect of BCG and non-tuberculous mycobacteria?. Int J Tuberc Lung Dis.

[5] Lee E, Holzman RS (2002). Evolution and current use of the tuberculin test. Clin Infect Dis.

[6] Huebner RE, Schein MF, Bass JB (1993). The tuberculin skin test. Clin Infect Dis.

[7] Nayak S, Acharjya B (2012). Mantoux test and its interpretation. Indian Dermatol Online J.

[8] Mazurek GH, Jereb J, Lobue P, Iademarco MF, Metchock B, Vernon A (2005). Guidelines for using the QuantiFERON-TB Gold test for detecting Mycobacterium tuberculosis infection, United States. MMWR Recomm Rep.

[9] Mazurek GH, Jereb J, Vernon A, LoBue P, Goldberg S, Castro K (2010). Updated guidelines for using interferon gamma release assays to detect Mycobacterium tuberculosis infection - United States, 2010. MMWR Recomm Rep.

[10] Menzies D, Pai M, Comstock G (2007). Meta-analysis: new tests for the diagnosis of latent tuberculosis infection: areas of uncertainty and recommendations for research. Ann Intern Med.

[11] Pollock NR, Campos-Neto A, Kashino S, Napolitano D, Behar SM, Shin D (2008). Discordant QuantiFERON-TB Gold test results among US healthcare workers with increased risk of latent tuberculosis infection: a problem or solution?. Infect Control Hosp Epidemiol.

[12] Talati NJ, Seybold U, Humphrey B, Aina A, Tapia J, Weinfurter P (2009). Poor concordance between interferon-gamma release assays and tuberculin skin tests in diagnosis of latent tuberculosis infection among HIV-infected individuals. BMC Infect Dis.

[13] Chkhartishvili N, Kempker RR, Dvali N, Abashidze L, Sharavdze L, Gabunia P (2013). Poor agreement between interferon-gamma release assays and the tuberculin skin test among HIV-infected individuals in the country of Georgia. BMC Infect Dis.

[14] Connell TG, Ritz N, Paxton GA, Buttery JP, Curtis N, Ranganathan SC (2008). A three-way comparison of tuberculin skin testing, QuantiFERON-TB gold and T-SPOT.TB in children. PLoS One.

[15] Swindells JE, Aliyu SH, Enoch DA, Abubakar I (2009). Role of interferon-gamma release assays in healthcare workers. J Hosp Infect.

[16] Szklo M, Nieto FJ (2014). Epidemiology: beyond the basics.

[17] Hashemi Shahri M, Fallah Ghajary A, Ansari Moghaddam A, Khadem Sameni F, Fayyaz Jahani F, Ahmadnezhad E (2012). To compare the performance of Quanti-FERON with the tuberculin skin test for identifying latent tuberculosis infection. Iran J Epidemiol.

[18] Mirhaghani L, Nasehi M (2002). National tuberculosis program in Iran.

[19] Khazaei HA, Rezaei N, Bagheri GR, Dankoub MA, Shahryari K, Tahai A (2005). Epidemiology of tuberculosis in the Southeastern Iran. Eur J Epidemiol.

[20] Rafiee S, Besharat S, Jabbari A, Golalipour F, Nasermoaadeli A (2009). Epidemiology of tuberculosis in northeast of Iran: a population-based study. Iran J Med Sci.

[21] Sofian M, Zarinfar N, Mirzaee M, Nejad SA (2009). Epidemiology of tuberculosis in Arak, Iran. Faslnamahi Kumish.

[22] Sim J, Wright CC (2005). The kappa statistic in reliability studies: use, interpretation, and sample size requirements. Phys Ther.

[23] Tripodi D, Brunet-Courtois B, Nael V, Audrain M, Chailleux E, Germaud P (2009). Evaluation of the tuberculin skin test and the interferon-gamma release assay for TB screening in French healthcare workers. J Occup Med Toxicol.

[24] Ozdemir D, Annakkaya AN, Tarhan G, Sencan I, Cesur S, Balbay O (2007). Comparison of the tuberculin skin test and the quantiferon test for latent Mycobacterium tuberculosis infections in health care workers in Turkey. Jpn J Infect Dis.

[25] Jo KW, Hong Y, Park JS, Bae IG, Eom JS, Lee SR (2013). Prevalence of latent tuberculosis infection among health care workers in South Korea: a multicenter study. Tuberc Respir Dis (Seoul).

[26] Dorman SE, Belknap R, Graviss EA, Reves R, Schluger N, Weinfurter P (2014). Interferon-γ release assays and tuberculin skin testing for diagnosis of latent tuberculosis infection in healthcare workers in the United States. Am J Respir Crit Care Med.

[27] Doosti-Irani A, Ayubi E, Mostafavi E (2016). Tuberculin and QuantiFERON-TB-Gold tests for latent tuberculosis: a meta-analysis. Occup Med (Lond).

